# Supplementary material for: Non-compliance with clinical guidelines increases the risk of complications after primary total hip and knee joint replacement surgery
Source: PLoS One. 2021 Nov 18;16(11):e0260146. doi: 10.1371/journal.pone.0260146 (PMC8601457; doi:10.1371/journal.pone.0260146)
Supplement: S1 File — (DOCX) [file pone.0260146.s001.docx]

## Supplementary File 1: Additional results

Supplementary Table 1: Information on prophylactic antibiotics

| Antibiotic prophylaxis | Yes (N, %) |
| --- | --- |
| Cephazolin | 1701 (90.7%) |
| Flucloxacillin | 84 (4.5%) |
| Vancomycin  Appropriately used vancomycin (used with patient indications) | 136 (7.3%)  43 (2.3%) |
| Gentamicin | 855 (45.6%) |
| Aztreonam | 0 |
| Fluroquinolone | 0 |
| Clindamycin | 9 (0.5%) |
| Cephalothin sodium | 218 (11.6%) |
| Ceftriaxone | 22 (1.2%) |
| Lincomycin | 15 (0.8%) |
| Cefotaxime | 2 (0.1%) |
| Piperacillin/tazobactam | 1 (0.1%) |

Supplementary Table 2: Number prophylactic medications taken

| **Number of medications** | **VTE chemical prophylaxis** | **Antibiotic prophylaxis** |
| --- | --- | --- |
| 0 | 7 | 0 |
| 1 | 1112 | 861 |
| 2 | 696 | 860 |
| 3 | 58 | 153 |
| 4 | 2 | 1 |

Supplementary Table 3: Information on chemical and mechanical prophylaxis

| **Chemical VTE prophylaxis** | **Pre-operative use** | **VTE prophylaxis /**  **post-operative use** |
| --- | --- | --- |
| Enoxaparin sodium | 13 (0.6%) | 1,078 (57.5%) |
| Dalteparin | 0 | 396 (21.1%) |
| Fondaparinux | 0 | 0 |
| Aspirin | 469 (25%) | 878 (46.8%) |
| Rivaroxaban | 13 (0.7%) | 161 (8.6%) |
| Warfarin | 68 (3.6%) | 78 (4.2%) |
| Heparin / unfractionated heparin | 4 (0.2%) | 74 (3.9%) |
| Dabigatran etexilate | 9 (0.5%) | 11 (.6%) |
| Apixaban | 2 (0.1%) | 10 (0.5%) |
| **Mechanical prophylaxis (N=1847)** |  | **VTE prophylaxis /**  **post-operative use** |
| Foot pumps |  | 277 (14.8%) |
| Calf compressors |  | 1415 (75.5%) |
| Graduated compression stockings |  | 1409 (75.1%) |
| Total number of people who used any mechanical prophylactic device |  | 1847 (98.6%) |

Supplementary Table 4: VTE Complications up to one-year post surgery

| **Type of VTE complications (N=160)** | **1^st^ VTE event** | **2^nd^ VTE event** | **3^rd^ VTE event** |  |
| --- | --- | --- | --- | --- |
| PE and DVT (0) | 2 | 2 | 0 |  |
| PE only (1) | 19 | 2 | 0 |  |
| Symptomatic DVT (2) | 34 | 1 | 1 |  |
| Asymptomatic DVT (3) | 21 | 1 | 0 |  |
| TOTALS | 76 (40.5%) | 6 (0.32%) | 1 (0.05%) |  |
| **Timeframe for VTE complications (N=160)** | **1^st^ VTE event** | **2^nd^ VTE event** | **3^rd^ VTE event** | **All VTE events** |
| During acute admission | 39 (2.1%) | 0 | 0 | 39 (2.1%) |
| Between acute discharge and 35 days | 25 (1.3%) | 5 (0.3%) | 0 | 30 (1.6%) |
| Between 36-90 days | 6 (0.3%) | 1 (0.1%) | 1 (0.1%) | 8 (0.4%) |
| Between 91-365 days | 6 (0.3%) | 0 | 0 | 6 (0.3%) |
| TOTALS | 76 (4.1%) | 6 (0.3%) | 1 (0.1%) | 83 (4.4%) |

Supplementary Table 5: Type of complications requiring hospital readmission

| **Reason for readmissions to hospital for surgical complications** | **1^st^ readmission (N=160)** | **2^nd^ readmission**  **(N=22)** | **3^rd^ readmission (N=3)** |  |
| --- | --- | --- | --- | --- |
| **All joint-related readmissions (N=1875)** | 136 (7.3%) | 20 (1.1%) | 3 (0.2%) |  |
| PE | 7 (0.4%) | 2 (0.1%) | 0 |  |
| DVT | 2 (0.1%) | 0 | 0 |  |
| Deep joint infection (reoperation) | 14 (0.7%) | 4 (0.2%) | 2 (0.1%) |  |
| Joint/wound Infection (readmitted and IV antibiotics) | 23 (1.2%) | 2 (0.1%) | 0 |  |
| Wound / joint /operated leg Bleeding | 4 (0.2%) | 0 | 0 |  |
| Joint Dislocation | 8 (0.4%) | 2 (0.1%) | 0 |  |
| Wound dehiscence | 2 (0.1%) | 0 | 0 |  |
| Severe joint stiffness | 50 (2.7%) | 7 (0.4%) | 1 (0.1%) |  |
| Other joint related reason | 26 (1.4%) | 3 (0.2%) | 0 |  |
| All non-joint related readmissions** | 24 (1.3%) | 2 (0.11%) | 0 |  |
| **Time period for hospital readmissions** | **1^st^ readmission** | **2^nd^ readmission** | **3^rd^ readmission** | |
| Acute discharge – 35 days | 75 | 4 | 0 | |
| 36 – 90 days | 43 | 7 | 1 | |
| 91 – 365 days | 42 | 11 | 2 | |
| TOTALS | 160 | 22 | 3 | |

*See further details non-joint related complications in Supplementary Table 7

Supplementary Table 6: Type of complications requiring reoperation

| **Reoperations for surgical complications** | **1^st^ reoperation**  **(N=107)** | **2^nd^ reoperation**  **(N=20)** | **3^rd^ reoperation**  **(N=6)** | **4^th^ reoperation**  **(N=2)** |
| --- | --- | --- | --- | --- |
| All Joint-related re-operations | 94 (%) | 17 (1.0%) | 6 (0.3%) | 2 |
| Joint revision | 9 (0.5%) | 5 (0.3%) | 1 (0.1%) | 2 (0.1%) |
| Joint Wash out | 14 (0.7%) | 4 (0.2%) | 4 (0.2%) |  |
| Manipulation under anaesthetic (MUA) | 48 (2.6%) | 5 (0.3%) | 1 (0.1%) |  |
| Open reduction of dislocated joint | 0 | 2 (0.1%) | 0 |  |
| Closed reduction of dislocated joint | 7 (0.4%) | 0 | 0 |  |
| Repair wound dehiscence | 3 (0.2%) | 0 | 0 |  |
| Exploratory joint surgery e.g. severe pain | 0 | 0 | 0 |  |
| Surgical management of bleeding | 3 (0.2%) | 0 | 0 |  |
| Other type of joint related reoperation | 4 (0.2%) | 1 (0.1%) | 0 |  |
| Joint specific soft tissue repair | 6 (0.3%) | 0 | 0 |  |
| Surgery for non-joint related complications* | 13 (0.7%) | 3 (0.1%) | 0 | 0 |
| **Time period for hospital readmissions** | **1^st^ reoperation** | **2^nd^ reoperation** | **3^rd^ reoperation** | **4^th^ reoperation** |
| During acute admission | 28 (1.5%) | 5 (0.3%) | 0 | 0 |
| Acute discharge – 35 days | 27 (1.4%) | 4 (0.2%) | 0 | 0 |
| 36 – 90 days | 40 (2.1%) | 8 (0.4%) | 3 (0.2%) | 1 |
| 91 – 365 days | 11 (0.6%) | 3 (0.1%) | 3 (0.2%) | 1 |
| TOTALS | 107 (5.7%) | 20(1.0%) | 6 (0.3%) | 2 (0.1%) |

*See further details non-joint related complications in Supplementary Table 7

Supplementary Table 7: Non-joint reasons readmission and reoperation

| **Non-joint related complications 0-35 days** (N=1875) | **Requiring readmission after acute discharge** | **Requiring reoperation (either during acute admission or requiring readmission)** |
| --- | --- | --- |
| Cardiac | 2 | 5 |
| Non-joint bleeding | 7 | 2 |
| Non-joint infection | 12 | 0 |
| Non-joint fracture | 1 | 1 |
| Gastrointestinal | 1 | 2 |
| Urinary retention/bladder | 2 | 2 |
| Epidural blood patch | 0 | 4* |
| Adverse drug reaction | 1 | 0 |
| TOTALS | 26 (1.39%) | 16 (0.85%) |

*2 people had 2 procedures

Supplementary Table 8: Type of bleeding complications up to one year after surgery (non-joint related up to 35 days)

| **Type of bleeding complications (1) (N=)** | **Joint related bleeding** | **Non-joint related bleeding** |
| --- | --- | --- |
| Type 1 (no healthcare action) | 9 (0.5%) | 1 (0.1%) |
| Type 2 (overt bleed, healthcare provided) | 39 (2.1%) | 8 (0.4%) |
| Type 3 (major intervention, readmission/reoperation) | 7 (0.4%) | 10 (0.5%) |
| Type 5 (fatal bleeding) | 0 | 0 |
| TOTALS | 55 (3.0%) | 19 (1.0%) |
| **Timeframe for bleeding complications (N=160)** | **Joint related bleeding** | **Non-joint related bleeding** |
| During acute admission | 38 (2.0%) | 8 (0.4%) |
| Between acute discharge and 35 days | 16 (0.9%) | 11 (0.6%) |
| Between 36-90 days | 2 (0.2%) | N/A |
| Between 91-365 days | 0 | N/A |
| TOTALS | 56* (3.0%) | 20** (1.1%) |

*included one person who experienced two bleeding events: one during acute admission and another between 36-90 days (both type 2)

**Included one person who experienced two bleeding events: one during acute admission and another between acute discharge and 35 days (type 1 and 3)
