# Supplementary material for: Non-compliance with clinical guidelines increases the risk of complications after primary total hip and knee joint replacement surgery
Source: PLoS One. 2021 Nov 18;16(11):e0260146. doi: 10.1371/journal.pone.0260146 (PMC8601457; doi:10.1371/journal.pone.0260146)
Supplement: Attachment — Submitted filename: Response to Reviewers_Badge_PONE-D-18-18793_26_03_2021_FINAL.pdf [file pone.0260146.s002.pdf]

Whitlam Orthopaedic Research Centre
Orthopaedic Department
Locked Bag 7103 Liverpool BC NSW 1871
T: +61 2 8738 9255   F: +61 2 9602 7187

26th March 2021

Dr Daniel Pérez-Prieto
Academic Editor
PLOS ONE

Dear Dr Pérez-Prieto,

RE: PONE-D-18-18793
Clinical guidelines: Does non-compliance increase risk of complications after total hip and knee joint replacement surgery?

Thank you for your further consideration of our extensively revised manuscript. The methods and scope of analyses is substantially different to the initial manuscript we submitted and provided the rationale for withdrawing our initial manuscript and seeking a review at PLOS Medicine. The manuscript has been checked for typographical and grammatical errors. The file names have been checked to ensure compliance with PLOS ONE style requirements.

This letter describes our response to the issues identified in the initial review. I have attached annotated copies of the initial manuscript submitted to PloS One (2018) and the subsequent manuscripts submitted to PLOS Medicine and transferred to PLOS One (2021) to highlight the changes compared to the current submission. We have also made some further revisions to fully address feedback from the previous review, and a tracked changes and clean version of the manuscript we would hope undergoes further review are also attached. As requested, I have provided responses in relation to the specific questions and issues raised:

1. Is the manuscript technically sound, and do the data support the conclusions?
The manuscript must describe a technically sound piece of scientific research with data that supports the conclusions. Experiments must have been conducted rigorously, with appropriate controls, replication, and sample sizes. The conclusions must be drawn appropriately based on the data presented. Reviewer: No

We believe that the revised manuscript describes a technically sound piece of scientific research with data that supports the conclusions. Based on the feedback provided by the PLOS ONE reviewers we have completed new analyses of the data and adjusted the statistical methods to increase the rigour of the data analyses.

In our initial submission, our conclusion was that there was no significant impact of non-compliance on overall composite outcome. The magnitude of effect for the primary outcome comprising composite surgical complications was 13% in the non-compliant group versus 8% in the group that received care considered compliant with both the guidelines for the prevention of VTE and antibiotic prophylaxis. This difference did not reach statistical significance. In this iteration we have also included new analyses examining the association between VTE compliance and VTE outcomes, and antibiotic compliance and

infection outcomes, both of which were statistically significant. We believe these additional analyses strengthen the scientific merit of the manuscript and clinical relevance of the results.

The revised methods now include:
"We conducted multiple logistic regression analyses to explore associations between non-compliance and risk of outcomes as follows: (1) non-compliance with both VTE and antibiotic guidelines and composite outcomes (2) VTE non-compliance and VTE outcomes, (3) antibiotic compliance with any SSI and (4) antibiotic compliance with deep SSI. Factors identified on univariate analysis with a p-value < 0.25 were entered into a backwards, stepwise multivariable logistic regression model (using the Akaike information criterion – AIC and forcing the main predictor, compliance, into the model) to identify independent risk factors associated with the relevant outcome for each analysis.

Missing data were imputed using multivariate imputation by chained equations (MICE). Model selection was performed using one of the imputed datasets. Effect estimates were taken from the pooled estimates using the five imputed datasets.

Sensitivity analyses were performed using complete case analysis and Bayesian information criterion (BIC). Further sensitivity analyses were completed without including routine doppler ultrasound (DUS) given this may mediate VTE complication outcomes. The full R code for all analyses are included in a supplementary file (Supplementary File 2)."

2. Has the statistical analysis been performed appropriately and rigorously? Reviewer: No

We have completed new and more extensive analyses in light of the reviewers' feedback as outlined in the methods above. The action taken for each suggestion are reported in more detail in our responses to the reviewers specific comments further below under General Comments.

3. Have the authors made all data underlying the findings in their manuscript fully available?

We initially indicated that data from this study are available upon request. We have clarified approval to make a deidentified data set including individual patient level data publicly available with the Lead Human Research Ethics Committee. We have uploaded this deidentified dataset to UNSW Data Archive and it will be published and publicly available via Research Data Australia https://researchdata.edu.au/ . The DOI for the data is still pending and was due to be confirmed on 18/03/2021. We can confirm this once known.

We believe the provision of this data is consistent with the PLOS Data policy, and should enable readers and reviewers to validate any of the results provided. Thank you for updating the Data Availability statement on our behalf to reflect the revised information that we have provided.

4. Is the manuscript presented in an intelligible fashion and written in standard English? Yes.
We have edited the revised manuscript to ensure a high standard of academic writing, appropriate use of grammar, and adherence to the required refencing style.

5. General comments:

Reviewer #1: The authors present the results of an observational cohort study in around 1875 patients with total hip and total knee replacement (THR, TKR) to investigate the compliance with clinical guidelines for prevention of complications. The primary endpoint was a composite comprising all-cause mortality, any VTE and any reoperation or readmissions within 35 days for medical issues or

within 365 days for joint related complications. The study shows higher rate of complications in the non-compliant group.

I first was very confused by the abstract, where no clear specification of the study type is given. Thus the sampling as well as the potential conclusions could not be followed.
To my understanding in the paper the authors conducted a prospective multicentric cohort study to investigate the compliance to clinical guidance for VTE as well as Antibiotic prophylaxis on the composite primary endpoint.

Revision to Abstract
The abstract has been revised to provide clearer information about the type of study conducted. This has included revision of the methods used to improve understanding of the sampling and potential conclusions. The reviewer correctly identified that this was a prospective multi-centre cohort study of people undergoing primary THR or TKR that aimed to determine the degree to which current practice in Australian hospitals was compliant with Australian guidelines to prevent VTE and infection after TKR or THR; and whether non-compliance with these guidelines is associated with increased risk of complications. The current iteration also has the results of the additional analyses.

The abstract now reports:

"Methods and Findings

A prospective multi-centre cohort study was undertaken in consenting adults with osteoarthritis undergoing primary TKR/THR at one of 19 high-volume Australian public or private hospitals."

1. **Individual data should be made available according to PLOS philosophy.**

As reported above a deidentified dataset has been uploaded to the UNSW Data Archive and it will be published and publicly available via Research Data Australia https://researchdata.edu.au/ . The DOI for the data is still pending and was due to be confirmed on 18/03/2021. We can confirm this once known.

2. **Taking into account the sample size argumentation, the general setting of the trial assumes an unadjusted effect. To me it is not clear, whether this is reasonable.**

In the initial submission we accounted for the impact of confounders in both the sample size calculations and in our analyses:

"Sample size calculation

Based on a previous study (31), the a priori compliance to non-compliance ratio in these participants was taken to be 2:1 (i.e. 67% versus 33%), and the prevalence of the composite outcome to be 7% in the compliance subgroup (reference group).

Calculation determined that 1102 participants in the compliant group and 551 participants in the non-compliant group (1653 in total) would provide 80% power at 5% significance level to detect a significant Relative Risk (RR) of 1.6 for non-compliance. This equated to a prevalence of the composite outcome of 11% in the non-compliant group compared to 7% in the compliant group. The planned sample size was increased to 2200 to consider a multiple correlation coefficient of 0.1 among known confounders, and to allow for 7% loss to follow-up by 35 days."

These calculations were informed by the following study:

Bozic KJ, Maselli J, Pekow PS, Lindenauer PK, Vail TP, Auerbach AD. The influence of procedure volumes and standardization of care on quality and efficiency in total joint replacement surgery. *J Bone Joint Surg Am.* 2010;92(16):2643-52. Epub 2010/11/19. doi: 10.2106/jbjs.i.01477. PubMed PMID: 21084575.

We used the above reference to estimate the expected levels of compliance for the a priori sample size calculations . Our expected event rate of 12.7% in the non-compliant group compared to 8.3% in the compliant group was close to the values used in our a-priori calculation (7% event rate in the compliant group). However, our compliance rate was very different. We anticipated a 67% compliance, yet reported only 4.5% compliance with both guidelines, 13.2% with the antibiotic guideline and 35.3% with the VTE guideline.

Section Sample Size: Please specify the test procedure used for sample size calculation. Give references to the assumed RR effect and the software used. The sample size calculation is based on a pooled analysis in particular without adjustment for effect modifiers.

The a priori power calculations were performed using the '**procpower**' calculation in SAS software for comparing two independent proportions. We used the reference noted above (Bozic et al., 2010) to the manuscript as the source used to estimate the potential prevalence of composite compliance and complications in our data set.

A adjusted analysis should be conducted using first main effects in the analysis. Hereby a suitable bivariate variable selection technique should be used, e.g. by fitting bivariate logistic regression models to the data and decide for variable inclusion by p-values below 25%. Interaction effects should be investigated as well. Results of this investigation have to be presented.

In this current iteration we performed separate unadjusted analyses for the outcomes of each analysis:

1. Composite non-compliance and composite surgical complications.
2. VTE non-compliance and VTE complications.
3. Antibiotic non-compliance and surgical site infection (SSI).
    3.1 Any SSI (requiring oral or IV antibiotics, hospital readmission or reoperation)
    3.2 Deep SSI only (requiring IV antibiotics, hospital readmission or reoperation)

"We conducted multiple logistic regression analyses to explore associations between non-compliance and risk of outcomes as follows: (1) non-compliance with both VTE and antibiotic guidelines and composite outcomes (2) VTE non-compliance and VTE outcomes, (3) antibiotic compliance with any SSI and (4) antibiotic compliance with deep SSI. Factors identified on univariate analysis with a p-value < 0.25 were entered into a backwards, stepwise multivariable logistic regression model (using the Akaike information criterion – AIC) to identify the association between guidelines compliance and complication outcomes for each analysis. We reported the final model after backwards stepwise regression using AIC and forcing only the main predictor (non-compliance) into each model."

In the initial submission we had performed a bivariate logistic regression although used a theoretical rather than an analytic approach as the reviewer has recommended. There are many possible ways to select possible confounders and in the initial submission we used clinical expertise of the authors and the literature to identify the confounders we included in the model. In this current iteration we have used a bivariate variable selection technique by fitting bivariate logistic regression models to the data and decide for variable inclusion by p-values below 25%, for each of the models .

The results of this analyses can be obtained from the code, and we have provided the eligible covariates included in the three models based on this analytical method (prior to backward stepwise regression).

Table: Factors included in adjusted regression models

**Model 1**	**Model 2**	**Model 3.1**
**Primary outcomes**		
Composite surgical complications	VTE complications	Surgical site infection complications
**Eligible covariates included**		
• **Composite Non compliance**<br>• Joint<br>• Routine doppler performed<br>• Cement fixation<br>• First day mobilised day 0 or 1<br>• Surgical drain used<br>• Body Mass Index (BMI)<br>• Age<br>• Preoperative comorbid<br>- kidney disease<br>- heart disease<br>- GORD<br>- Stroke<br>- Hypertension<br>- Sleep apnoea<br>- Hypercholesterolaemia<br>- Lung disease<br>- Past history cancer<br>- Musculoskeletal disease<br>- Previous hip replacement<br>• Blood transfusion received<br>• Surgical duration<br>• ASA score<br>• Preoperative medications for pain included:<br>- anticonvulsant / antidepressant<br>- non-steroidal anti-inflammatory<br>- Yes / no (took any)	• **VTE Non compliance**<br>• Antibiotic Non compliance<br>• Joint<br>• Routine doppler performed<br>• Cement fixation<br>• First day mobilised day 0 or 1<br>• Surgical drain used<br>• Body Mass Index (BMI)<br>• Age<br>• Preoperative comorbid<br>- kidney disease<br>- heart disease<br>- GORD<br>- Stroke<br>- Hypertension<br>- Sleep apnoea<br>- Hypercholesterolaemia<br>- Lung disease<br>- Past history cancer<br>- Musculoskeletal disease<br>- Depression and/or anxiety<br>- History VTE<br>- Previous hip replacement<br>• Blood transfusion received<br>• Surgical duration<br>• ASA score<br>• Preoperative medications for pain included:<br>- anticonvulsant / antidepressant<br>- non-steroidal anti-inflammatory<br>• Current smoker	• **Antibiotic Non compliance**<br>• VTE Non compliance<br>• Joint<br>• Routine doppler performed<br>• Cement fixation<br>• Surgical drain used<br>• Body Mass Index (BMI)<br>• Age<br>• Preoperative comorbid<br>- kidney disease<br>- heart disease<br>- GORD<br>- Stroke<br>- Hypertension<br>- Sleep apnoea<br>- Hypercholesterolaemia<br>- Lung disease<br>- Past history cancer<br>- Musculoskeletal disease<br>- Diabetes<br>- Neurological condition<br>- History VTE<br>- Previous hip replacement<br>• Blood transfusion received<br>• Surgical duration<br>• ASA score<br>• Preoperative medications for pain included:<br>- anticonvulsant / antidepressant<br>- non-steroidal anti-inflammatory<br>- opioids<br>• Current smoker<br>• Neuraxial anaesthesia<br>• Level of education<br>• Public / private hospital<br>• Bilateral joint replacement

Interaction effects should be investigated as well. Results of this investigation have to be presented.

We tested each of the four models with all two-way interaction terms between the variables retained in the final model after backwards stepwise regression, and the relevant primary predictor for each model:

1. Composite non-compliance
2. VTE non compliance

3.1. Antibiotic non-compliance (all SSI model)

3.2. Antibiotic non-compliance (deep SSI model)

None of the interaction terms were significant in any model. Note that the primary predictor was only significant for analyses 2 and 3.1.

We included the following information in the manuscript to describe the methods and results of these analyses:

Methods: *Interaction terms for the main predictor (non-compliance) against each other variable were tested in the final mode for each analysis.* (See manuscript 11-12)

Results: *There was no significant interaction between the main predictor (non-compliance) and other variables included in the final model and the other three analyses completed.* (See manuscript 19 and 21)

The effect of missing data on the results should be elaborated, e.g. by multiple imputation techniques.

The level of missing data was low, although we have now completed a more robust method using multivariate imputation by chained equations (MICE) to consider the impact of missing data for each of the four adjusted models. There were no significant differences between models using imputed versus complete case analysis. Imputed data were used for the primary analyses; compete case analysis was performed as part of the sensitivity analyses.

"Missing data were imputed using multivariate imputation by chained equations (MICE). Model selection was performed using one of the imputed datasets.

Effect estimates were taken from the pooled estimates using the five imputed datasets. We tested the models with all two-way interaction terms entered. None of these was significant. Sensitivity analyses were performed using complete case analysis and Bayesian information criterion (BIC). Further sensitivity analyses were completed without including routine doppler ultrasound (DUS) given this may mediate VTE complication outcomes. The full R code for all analyses are included in a supplementary file (Supplementary File 2)."

Analysis of secondary endpoints related to the guideline compliance are missing.

In the current iteration of this manuscript, we have included separate analyses looking at specific outcomes related to VTE and to infection as follows.

1. Composite non-compliance and other potential confounding and mediating factors in the dataset and composite surgical complications.
2. VTE non-compliance and other potential confounding and mediating factors in the dataset and VTE complications.
3. Antibiotic non-compliance and other potential confounding and mediating factors in the dataset and surgical site infection complications.

3.1 Any SSI (requiring oral or IV antibiotics, hospital readmission reoperation)
3.2 Deep SSI only (requiring IV antibiotics, hospital readmission reoperation)

We have examined complications up to one year to support maximal event rate included in analyses. The new supplementary tables 3-8 provide additional information about complication outcomes at the three follow-up times (35, 90 and 365 days post surgery).

<u>Supplementary File 1: Additional results (Word document)</u>
Supplementary Table 4: VTE Complications up to one-year post surgery
Supplementary Table 5: Type of complications requiring hospital readmission
Supplementary Table 6: Type of complications requiring reoperation
Supplementary Table 7: Non joint reasons readmission and reoperation
Supplementary Table 8: Type of bleeding complications up to one-year post surgery (non-joint related up to 35 days)

I would be happy to provide further information if required to support further review of the revised manuscript. I look forward to further feedback about this manuscript.

Yours sincerely

Helen Badge
PhD Candidate, UNSW / Whitlam Orthopaedic Research Centre
Lecturer, Australian Catholic University
E: helen.badgehawke@gmail.com

Attachments:
<u>Current manuscript for further review</u>
1. Badge et al, EPOC manuscript_PLoS One_26032021_tracked (tracked changes)
2. Badge et al, EPOC manuscript_PLoS One_26032021_clean (clean)

<u>Manuscript submitted to PLoS Medicine and transferred to PLos One (2021):</u>
3. Badge et al, EPOC annotated manuscript_PLoS One_23022021 (annotated to highlight changes from initial submission)

<u>Manuscript submitted to PLos One (2018):</u>
4. Badge_PLOSOne_Annotated 2018 Manuscript_initial submission (annotated to highlight changes from initial submission)

<u>Statistical analyses R code files</u>
5. Badge, EPOC, Supplementary file 1, Main analysis_26032021
6. Badge, EPOC_Supplementary file 2, Supp code_PLoS Medicine
